# Phytochemical Profiles and In Vitro Immunomodulatory Activity of Ethanolic Extracts from *Galium aparine* L.

**DOI:** 10.3390/plants8120541

**Published:** 2019-11-25

**Authors:** Tetiana Ilina, Natalia Kashpur, Sebastian Granica, Agnieszka Bazylko, Igor Shinkovenko, Alla Kovalyova, Olga Goryacha, Oleh Koshovyi

**Affiliations:** 1 National University of Pharmacy, 53-Pushkinska str., 61002 Kharkiv, Ukraine; ilyinatany86@gmail.com (T.I.); shinkovenko@mail.ru (I.S.); allapharm@yahoo.com (A.K.); helgagnosy@gmail.com(O.G.); oleh.koshovyi@gmail.com (O.K.); 2 Mechnikov Institute of Microbiology and Immunology, National Academy of Medical Sciences of Ukraine, 14/16-Pushkinska str., 61057 Kharkiv, Ukraine; natali.kashpur@ukr.net; 3 Department of Pharmacognosy and Molecular Basis of Phytotherapy, Medical University of Warsaw, 1 Banacha str., 02-097 Warsaw, Poland; agnieszka.bazylko@wum.edu.pl

**Keywords:** *Galium aparine* L., ethanolic extracts, phenolic compounds, iridoids, immunomodulatory activity, lymphocyte blast transformation

## Abstract

*Galium aparine* L., family Rubiaceae, is a widely spread species in the *Galium* genus. The herb of *G. aparine* is part of folk remedies and dietary supplements. In this study, we analyzed the chemical composition and immunomodulatory activities of *G. aparine* herb ethanolic extracts obtained from the plant material by maceration with 20%, 60% or 96% ethanol. The contents of hydroxycinnamic acid derivatives, flavonoids and polyphenols were determined spectrophotometrically, with extractives and polysaccharides quantified gravimetrically. The qualitative composition was studied using UHPLC-DAD-MS/MS analysis; isolation not previously described in *G. aparine* quercetin rhamnoglucoside was carried out through column chromatography, and the immunomodulatory activity of extracts was determined in the reaction of lymphocyte blast transformation. Major constitutes of extracts were iridoids, i.e., monotropein, 10-desacetylasperulosidic acid and asperulosidic acid; *p*-hydroxybenzoic acid; hydroxycinnamic acid derivatives, i.e., 3-*O*-caffeoylquinic, 5-*O*-caffeoylquinic, 3,4-*O*-dicaffeoylquinic, 3,5-*O*-dicaffeoylquinic, 4,5-O-dicaffeoylquinic acids and caffeic acid derivatives; flavonoids, i.e., rutin, quercetin 3-*O*-rhamnoglucoside-7-*O*-glucoside, and isorhamnetin 3-*O*-glucorhamnoside. Significantly, quercetin 3-*O*-rhamnoglucoside-7-*O*-glucoside was first isolated and identified in *Galium* species so far investigated. All *G. aparine* herb ethanolic extracts stimulate the transformational activity of immunocompetent blood cells, with 96% ethanolic extract being the most active. The data obtained necessitate further research into the mechanisms of immunomodulatory activity of extracts from *G. aparine* herb.

## 1. Introduction

Disturbances of the immune system lead to the development and complications of chronic diseases. Numerous studies have proved that the restoration of immune system function is a prerequisite for the successful therapy of various illnesses [1,2]. The development of the immune response is the result of the cooperative impact T-, B-lymphocytes and macrophages, associated with activation, proliferation and differentiation of immunocompetent cells.

Specific immunostimulants include thymus preparations, interleukins, interferons, biologically active peptides, polysaccharides of certain fungi and therapeutic vaccines, whose effect is explained through their ability to influence the metabolism of cells and body tissues and activate immunocompetent cells.

Many plant-derived compounds, like sterols, polysaccharides, alkaloids, flavonoids, lectins and glycoprotein, are used for immunomodulation [3]. For example, among polysaccharides, acidic arabinogalactan and ramnogalacturonan have been shown to manifest immunostimulatory effect in vitro and in vivo [4]. Numerous studies look into the immunomodulative activities of saponins [5,6,7,8]. The proven effect of triterpenoid glycosides on the immune system of mammals contributed to the development of a wide range of dietary supplements for the prevention of the immune system disturbances, i.e., human immunity system enhancement [9], and for the prevention and treatment of allergies [10,11].

In our previous studies, the immunomodulative effect of the aqueous and ethanolic extracts of *Galium verum* L. herb was established [12,13]. Among other species of the genus *Galium* L., one of the most widely spread is *Galium aparine* L., also called cleavers or goosegrass, which can be found all over Ukraine, Europe, Northern America and certain parts of Asia; its habitat in the north reaches Alaska and Greenland whereas as introduced species it can be found in Australia, New Zealand and sub-Antarctic islands. Extensive research on the phytochemical composition of *G. aparine* showed that cleavers herb contains iridoids: asperulosidic acid and 10-deacetylasperulosidic acid [14], monotropein, asperuloside, acumine and aucubin [15]; alkaloids: protopine, harmine, (±)-vasicinone, (−)-l-hydroxypeganine and (−)-8-hydroxy-2,3-dihydrodesoxypeganine [16]; phenolcarbonic and hydroxycinnamic acids: chlorogenic, caffeic, *n*-coumaric, ferulic, caftaric and gentisic [17,18]; flavonoids: quercetin, dihydroquercetin, rutin, hyperosid, isoquercetrin, kaempferol, kaempferol-3-*O*-glucoside (astragalin), epicatechin, neohesperidin, luteolin, luteolin-7-*O*-diglucoside and apigenin [18,19,20,21]; cholesterol, campesterol, stigmasterol, sitosterol, **Δ**-[5]-avenasterol, **Δ**-[7]-stigmasterol and **Δ**-[7]-avenasterol [21,22,23]. According to our previous studies triterpenoids (oleanolic, ursolic, euscaphic and tormentic acids, betulin and lupeol), sesquiterpenoids, squalene, aromatic compounds and higher alkanes, and their derivatives fatty acids, chlorophylls, and carotenoids were identified in extracts of cleavers herb [24,25].

The extract from the herb of *G. aparine* is one of the ingredients of some galenic remedies and dietary supplement that are recommended as immunomodulatory, anti-inflammatory and for detoxication, as well as for the improvement of the functioning of the lymphatic and blood circulatory systems and a drainage drug, based on the activation of the immune system and normalization of impaired functions.

In the ethnopharmacology of many countries, the herb of *G. aparine* is used for treatment of skin diseases [26,27,28].

In the literature several biological activities of *G. aparine* are reported. Previous studies confirmed antimicrobial, antioxidant and anti-cancer effects of different extracts from this plant material [18,25,29]. Although the plant material is used as a potential immunomodulator there are no papers investigating this kind of bioactivity of *G. aparine*.

The aim of the present article is to investigate the chemical composition and immunomodulatory activity of different ethanolic extracts from *G. aparine* using the lymphocyte blast transformation model in vitro (RLBT).

## 2. Results and Discussion

### 2.1. Phytochemical Screening of G. aparine Herb Ethanolic Extracts

The phytochemical screening of *G. aparine* herb ethanolic extracts revealed the presence of polysaccharides only in Extract I (20% EtOH, *v*/*v*), whereas flavonoids (flavonols and flavones) and phenolic acid derivatives were detected and identified in all samples under study, and the results obtained correspond with previous studies [17,18,19,20,21,22].

### 2.2. Quantification of Main Groups of Phytochemicals in Analyzed Extracts

The content of the main groups of phytochemicals in *G. aparine* herb ethanolic extracts is given in Table 1 below.

A comparative study shows differences between the *G. aparine* herb ethanolic extracts investigated in the present research.

The highest extraction yield (252.7 mg/mL) was obtained when 20% ethanol was used (extract I), and the lowest (163.4 mg/mL) in the case of 96% ethanol extraction (extract III).

Comparable content of hydroxycinnamic acid derivates is marked in the extracts I and II, and the highest content in extract III. Flavonoid content is comparable in extracts I and III whereas it was significantly lower in extract II. Extracts I and III contain similar amounts of polyphenols while in extract II the content of this class of phytochemicals was significantly lower.

The presence of polysaccharides was confirmed only in extract I (129.4 mg/g). This observation is in the agreement with the general expectation that polysaccharides are easily extracted with a solvent mixture with a high water content and generally not present if extracts are prepared with polar organic solvents like ethanol.

The data obtained display some differences from those by other researchers [17,22], which may result from various factors, such as the growth conditions of the plants under study or the methods of extraction and analysis.

### 2.3. UHPLC-DAD-MS/MS Analysis 

**Figure 1:**
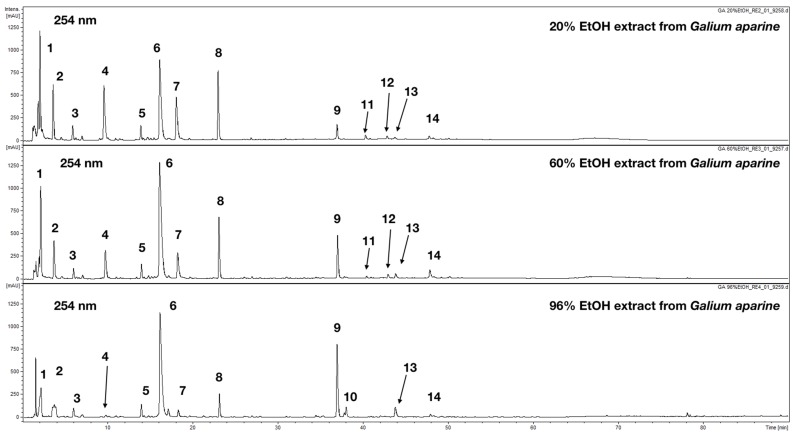
UHPLC-DAD-MS/MS chromatogram of *G. aparine* extracts recorded at 254 nm.

The chromatographic analysis of tested extracts was performed using a hyphenated chromatographic technique in order to characterize the phytochemical content of prepared extracts. In total 14 major constituents in three analyzed extracts (20% EtOH, 60%, EtOH and 96% EtOH) were detected and characterized (Figure 1, Table 2).

Compounds (**4**, **6**, **7**, **10** and **12–14**) exhibiting characteristic UV-Vis maxima at ca. 240 and 325 nm, with a shoulder at 300 nm, were characterized as caffeic acid derivatives. Based on observation of their fragmentation patterns and after comparison with chemical standards available they were identified as chlorogenic acids (4, 6, 7, M-H at *m*/*z* = 353 in MS-) and dicaffeoylquinic acids (12–14 M-H at *m*/*z* = 515 in MS^−^). Compound **10** present only in 96% EtOH extract was a caffeic acid derivative but further identification was not possible due to the lack of more data. Compounds **3** was identified as a simple phenolic acid (*p*-hydroxybenzoic acid).

Compounds **8**, **9** and **11** were identified as flavonoids with typical UV-Vis maxima observed at ca. 255, 260 and 350 nm. Compound **9** was easily elucidated (M-H at *m*/*z* 609 fragmented to 301 amu in negative ion mode) as popular quercetin derivative—rutin. This compound was previously described in investigated species and other belonging to the *Galium* genus [19]. Compound **11** showed a higher molecular mass with a pseudomolecular ion at *m*/*z* = 615 in MS^−^. The fragmentation showed easy cleavage of the methyl group M-H-15 at *m*/*z* = 609 and presence of aglycone moiety in fragmentation spectrum at *m*/*z* = 315. The comparison made with available chemical standards led to the identification of **11** as isorhamnetin 3-*O*-rhamnoglucoside. Compound **8** was also assigned as a quercetin derivative due to the presence of aglycone moiety ion at *m*/*z* = 301 in MS-. The molecular mass of 772 amu and fragmentation patterns in MS^−^ and MS^+^ led to the conclusion that this compound consists of quercetin linked to 2 hexose and 1 rhamnose units. The final identification required isolation and structure elucidation by 1H NMR.

Compounds **1**, **2** and **5** displayed a single absorption maximum at ca. 235 nm. Based on MS, spectrum molecular masses of 390 amu were confirmed for **1** and **2** and 432 amu for **5**. After literature research compounds were characterized as iridoid glycosides. Compound **1** and **2** showed the same molecular masses and they were tentatively assigned as monotropein and 10-desacetylasperulosidic acid, respectively. Both chemicals were previously detected in aerial parts of *G. rivale* and *G. mollugo*. Additionally, Compound **2** was reported from *G. album*. Compound **5** was assigned as asperulosidic acid. This natural product was found in aerial parts of *G. rivale*, *G. album* and *G. mollugo* [30,31,32].

To the best of our knowledge, apart from monotropein, 10-desacetylasperulosidic acid, asperulosidic acid (1, 2, 5), chlorogenic acid (6) and rutin (9), the presence of the rest of the natural products is reported in *G. aparine* for the first time.

### 2.4. Structure Elucidation of Isolated Quercetin Derivative 

Because the full elucidation of the chemical structure of Compound **8** was not possible based on UHPLC data obtained from performed analysis, this flavonoid derivative was isolated, and its chemical structure had to be confirmed using NMR analysis. The preliminary ^1^H analysis showed that **8** most probably is a quercetin derivative (it was also confirmed by the observation of its fragmentation pattern, Table 2). The ^13^C NMR spectrum displayed signals that were in the absolute agreement with previous report on flavonoid glycosides from *Ficaria verna* [33]. Finally, Compound **8** was identified as quercetin 3-*O*-rhamnoglucoside-7-*O*-glucoside. ^1^H and ^13^C NMR data of Compound **8** are given below.

*Quercetin 3-O-rhamnoglucoside-7-O-glucoside* (**8**)—yellow powder, ^1^H NMR (300 MHz, DMSO-d_6_) δ 7.56 (s, 1H), 7.54 (s, 1H), 6.85 (d, *J* = 8.9 Hz, 1H), 6.72 (d, *J* = 1.9 Hz, 1H), 6.44 (d, *J* = 1.9 Hz, 1H), 5.38 (d, *J* = 7.0 Hz, 1H), 5.07 (d, *J* = 7.2 Hz, 1H), 4.38 (s, 2H), 3.71 (d, *J* = 9.8 Hz, 2H), 3.14–3.01 (m, 3H), 1.00 (d, *J* = 6.1 Hz, 2H), ^13^C NMR (75 MHz, DMSO-d_6_) δ 177.54, 162.83, 160.90, 157.18, 156.01, 148.69, 144.81, 133.55, 121.67, 120.97, 116.44, 115.23, 105.62, 101.05, 100.73, 99.86, 94.52, 77.16, 76.39, 76.00, 74.06, 73.14, 71.83, 70.55, 70.36, 69.57, 68.24, 17.76.

It was detected for the first time in *G. aparine* and there are no reports on its occurrence in other *Galium* species.

### 2.5. In Vitro Reaction of Lymphocyte Blast Transformation 

The research presented is the first known study of the immunomodulatory activity of *G. aparine* herb ethanolic extracts.

It was established that all samples used in the current study considerably stimulate the transformational activity of peripheral blood mononuclear cells. Under the influence of the extracts used, 32.4–45.2% of mononuclear cells were involved in the proliferation process, which indicates the stimulating effect of the substances on T- and B-lymphocytes (Table 3), which is slightly lower than the level of PHA (phytohemagglutinin) preparation (Table 3).

Table 3 shows the summary of obtained results in the lymphocyte blast transformation model. The highest immunostimulatory activity was observed for 96% EtOH at concentrations of 0.74 and 1.47 mg/mL. The rest of the investigated samples displayed weaker activity than the positive control PHA.

The data obtained show that ethanolic extracts from *G. aparine* have an immunomodulatory potential. The highest activity in the investigated model was observed for 96% EtOH preparation (III). Based on phytochemical analysis it was shown that most probably polysaccharides that usually are suspected as the group of phytochemicals with immunostimulatory activity are not responsible for the observed results in the present study. Polysaccharides were detected and quantified only in 20% EtOH extracts, which did not display the best immunostimulatory potential (Table 1 and Table 3) as it would be expected, taking into account the general biological properties of plant polysaccharides. Interestingly the highest content of major phenolics detected, namely flavonoids and hydroxycinnamic derivates (Table 1), was observed for the 96% ethanolic extract. The UHPLC analysis revealed that the content of chlorogenic acid (**6**) increased (Table 2, from 18.44 to 40.61 µg/mg of dry extract) together with the ethanol percentage in the solvent used for extract’s preparation. The same pattern was observed for Compound **13** namely 3,5-*O*-dicaffeoylquinic acid (Table 2, 0.23 to 2.43 µg/mg). At the same time the content of other chlorogenic acid isomers (**4** and **7**) was decreasing (Table 2). In the case of flavonoids, the content in Compound **8** was lower in 96% EtOH extract (1.50 µg/mg) compared to 3.35 µg/mg in 20% EtOH extract. On the other hand, the rutin (**9**) content was raising with the EtOH percentage in the extraction medium (Table 1, from 1.08 to 6.33 µg/mg). The content of iridoids, which were not quantified in the present study seem to be decreasing based on the observation of peak, highs and areas in chromatograms acquired at 254 nm (Figure 1). The increasing content of phenolics (caffeic acid derivatives and flavonoids) in investigated extracts and lower the content of iridoids in 96% and 60% EtOH compared to 20% EtOH suggest that polyphenols contained in *G. aparine* may be responsible for the observed immunomodulatory activity. It is also possible that other more lipophilic compounds are present in analyzed extracts which were not detected with available methodology. The obtained results suggest significantly weaker activity of investigated extracts as immunostimulatory agents compared to previously reported results for extracts from *G. verum* [12].

## 3. Materials and Methods 

### 3.1. Plant Material

*G. aparine* herb was harvested at full flowering stage in the botanical garden of the National University of Pharmacy, Kharkiv, Ukraine, (geographic coordinates, latitude: 50°01′08.6″ N, longitude: 36°19′12.5″ E) in May, 2017. Voucher specimens no. 20052017–23052017 were deposited at the Department of Pharmacognosy (National University of Pharmacy, Ukraine). The identity of plants was established with the consulting assistance of T. Gontova, D.Sc. (Pharmacy), the Head of Department of Botany National University of Pharmacy [34].

### 3.2. Equipment

Spectrophotometer EvolutionTM 60S UV-Visible (Thermo Fisher Scientific, Waltham, MA, USA), Dionex Ultimate 3000RS system (Dionex, San Jose, CA, USA) coupled with an Amazon SL spectrometer, prep-HPLC—Shimadzu LC-20AP equipped with UV-Vis detector, sampler SIL-10A and fraction collector FRC-10 (all, Shimadzu, Kioto, Japan), Varian VNMRS 300 MHz spectrometer, electronic analytical scales AN 100 “Axis” (AXIS, Warszawa, Poland), electrical temperature chamber TC80M-3 (Medlabortekhnika, Ukraine), centrifuge OPN-3 (Phizpribor, Russia), microscope ZEISS Primo Star (ZEISS, Oberkochen, Germany), pipette Thermo Scientific, Lait series 1–200 μL (Thermo Fisher Scientific, Waltham, USA), pipette Thermo Scientific, Lait series 1–50 μL (Thermo Fisher Scientific, Waltham, USA), pipette Thermo Scientific, Lait series 1–1000 μL (Thermo Fisher Scientific, Waltham, MA, USA), pipette Thermo Scientific, Lait series 1–20 μL (Thermo Fisher Scientific, Waltham, MA, USA), CO_2_ incubator (Binder, Tuttlingen, Germany), bioanalyzer Agilent 2100 (Agilent, Santa Clara, CA, USA).

### 3.3. Chemicals

96% Ethanol (POCH, Gliwice, Poland) and purified water (produced by Merck Millipore, Simplicity UV system) used during extraction complied with the requirements of the State Pharmacopoeia of Ukraine [35]; chemicals for phytochemical screening: ethanol (POCH, Gliwice, Poland), DMSO, p.a. (POCH, Gliwice, Poland), hydrochloric acid, p.a. (Sobstar, Zaporizhia, Ukraine), acetic acid, puriss. (PJSC AZOT, Cherkasy, Ukraine), lead (II) acetate, p.a. (Unikhim Ltd., Novbosibirsk, Russia), aluminum chloride, p.a., granulated zinc, p.a. (PC Uralskiy zavod khimicheskih reaktivov, Moscow, Russia), ferric (III) chloride, puriss (Sigma-Aldrich, St. Louise, USA), gallic acid (Carl Roth, Karlsruhe, Germany) chlorogenic acid and rutin were of analytical grade (Merck Millipore, Burlington, MA USA); acetonitrile and formic acid for UHPLC was purchased from Sigma-Aldrich, St. Louise, USA.

### 3.4. Preparation of the Extracts 

As a solvent, ethanol at various concentrations (20%, 60% and 96%, *v*/*v*) was used; the extraction was carried out at a general ratio of the plant material solvent of 1:10 on heating with reflux. The extraction was repeated thrice under the same conditions (30 min each). The extracts obtained were combined and concentrated on a vacuum rotary evaporator to dryness at 45 °C. For all bioassays stock solutions of extracts (20 mg/mL) were prepared in DMSO-water 1:1 and diluted properly.

### 3.5. Preliminary Phytochemical Screening of G. aparine Herb Ethanolic Extracts

The preliminary phytochemical screening was performed using generally accepted methods and techniques of phytochemical analysis [36,37]. Polysaccharides were precipitated with three volumes of 96% ethanol. Glycosides and aglycons of flavonoids were determined in extracts from *G. aparine* herb in the reactions of identification: cyanidin reaction by Bryant (yellow-red colouring of the aqueous phase and yellow-hot colouring of the octal phase), the reaction with 3% solution of iron (III) chloride (dark green colour of flavonols, flavones); the reaction with an alkaline solution (bright yellow colour); the reaction with 5% solution of aluminium chloride (yellow-green colouring); the boric-acid reaction (yellow colouring on detection of 3- and 5-hydroxyflavones and 5-hydroxyflavanones); the reaction with ammonia (flavones, flavonols, flavanones and flavanonols dissolve with formation of yellow colour, which, when heated, changes to orange or brown colour).

To determine tannins, the reactions of the sediment were carried out with a 1% gelatine solution, a 1% solution of quinine hydrochloride and 10% solution of basic acetate of lead. The group of tannins was detected by the reaction with a solution of iron ammonium alum.

### 3.6. Quantification of the Main Groups of Phytochemicals

In all the extracts from *G. aparine* herb the extractive substances were determined gravimetrically [38]; polysaccharides were quantified gravimetrically after complete drying at room temperature taking into account the loss on drying; the sum of the hydroxycinnamic acid derivates was determined by direct spectrophotometry (as chlorogenic acid, λ = 325 nm) according to Yezerska et al. [39], Spagnol et al. [40]; flavonoids were quantified by the method of differential spectrophotometry with aluminium chloride (as rutin, λ = 410 nm) [41]; polyphenols were quantified by direct spectrophotometry (as gallic acid, λ = 270 nm) according to Koshovyi et al. [42]. All assays were performed in triplicate.

### 3.7. UHPLC-DAD-MS/MS Analysis

Extracts prepared from aerial parts of *G. aparine* were dissolved in methanol:water or water to obtain a final concentration 10 mg/mL. Samples were filtered through a 0.45 µm syringe filter and subjected to UHPLC-DAD-MS analysis using a Dionex Ultimate 3000RS system coupled with an Amazon SL spectrometer. The separation was carried out with a Kinetex XB-C_18_ column (150 mm × 2.1 mm × 1.7 µm, Phenomenex, USA) maintained at 25 °C. The flow rate was set at 0.3 mL/min. The mobile phase A was aqueous solution of formic acid (0.1%) and B was 0.1% HCOOH in acetonitrile. The following gradient elution was used: 0 min—4%B, 60 min—26%B and 90 min—95%B. A 3 µ extract solution was injected into the UHPLC column. UV-Vis spectra of detected compounds were recorded over the range from 190 to 450 nm. The chromatogram was acquired at 254 nm, 325 nm and 350 nm. Mass spectra were recorded in the negative and positive ion mode. Compounds were identified basing on their UV-Vis and MS spectra. Comparisons with the available chemical standards and suitable literature were performed [43,44,45,46]. Compounds were quantified using calibration curves for chlorogenic acid at 325 nm for all caffeic acid derivatives found in the extracts or using calibration curve for isoquercitrin for all flavonoids. Calibration curves were generated based on the amounts of compounds injected into UHPLC vs. peak areas. A linear fit was used for all calculations. Stock solution of chlorogenic acid and isoquecitrin was prepared in ethanol (final concentration was 50 µg/mL.

### 3.8. Isolation of Major Constituents of 96% EtOH Extract (Compounds 6, 8 and 9)

The isolation of major compounds detected in 96% EtOH extract was performed using prep-HPLC—Shimadzu LC-20AP equipped with UV-Vis detector, sampler SIL-10A and fraction collector FRC-10 (all, Shimadzu, Kioto, Japan). One gram of 96% EtOH extract was dissolved in DMSO and filtered through PVDF 5 µm syringe filter. Compounds were separated on Kinetex XB-C_18_ column (Phenomenex, USA, 150 mm × 22.1 mm × 5 µm) maintained at 25 °C. The flow rate was 20 mL/min. The mobile phase A was aqueous solution of formic acid (0.1%) and B was 0.1% HCOOH in acetonitrile. The following gradient elution was used: 0 min—2%B, 60 min—26%B and 90 min—95%B. Four hundred µL of dissolved raw extract was injected into the HPLC system ten times. Chromatogram was recorded at 254 nm. Compounds were collected at 18.6–19.5 min (6), 22.6–22.9 min (8) and 34.6–35.2 min (9). Obtained eluates were freeze dried to obtain 11 mg of 6, 4 mg of 8 and 7 mg of 9. The NMR spectra were recorded with Varian VNMRS 300 MHz spectrometer in DMSO-d6.

### 3.9. Study of Immunomodulatory Activity

To assess the immunomodulatory activity of the extracts obtained, in vitro RLBT with an adequate resolution was used [47,48].

As a sample for substance testing, the mononuclear cells (lymphocytes) removed from venous heparinized blood (donated blood, Kharkiv Regional Blood Banking Centre, UA) by Ficoll-verographine gradient density centrifugation (density 1.077 g/mL) (Research and Production Enterprise “PanEco”, Russia) by the standard technique [49], were used (Protocol of Committee on Biomedical Ethics of SO “Mechnikov Institute of Microbiology and Immunology” No. 8 of December 5, 2018). The study conformed to the principles of the Declaration of Helsinki.

The cells obtained were cultured in medium 199 with addition of 10% bovine fetal serum (Thermo Fisher Scientific, Waltham, MA, USA) and 2 mM L-glutamine (Altera Holding, RU), 100 μg/mL gentamicin (LEK (CZ). A suspension of 106 per 1 mL of the culture medium with the addition of substances was incubated for 15–18 h in a thermostat at 37 °C, in a 5% CO_2_ atmosphere with saturated water vapor.

The intensity of the proliferative reaction was evaluated by the indices of DNA (deoxyribonucleic acid) synthesis activation recorded by the treatment of samples with anti-BrdU (5-bromo-2′-deoxyuridine) Antibody (3H579) monoclonal antibodies (Santa Cruz Biotechnology, CA, USA) at the concentration of 100 mg/mL. After the final sample preparation, numerical data on the total number of cells and the percentage of blast forms in the samples were established for the flow cytometric analysis with fluorescence detection.

Before the RLBT, the stock solutions of analysed extracts were prepared at concentrations of 0.20, 1.48 and 3.48 mg/mL. One hundred μL of substances were added to 100 μL of primary cultures of immunocompetent cells to obtain final concentrations of 0.10, 0.74 and 1.74 mg/mL. The mitogenic stimulation of lymphocytes by PHA (Research and Production Enterprise “PanEco”, RU) at the concentration of 2.5 μg/mL was performed as a control. RLBT without the addition of the substances under study (spontaneous blast transformation) was also evaluated.

### 3.10. Statistical Analysis

All statistical analyses were carried out in accordance with the requirements of the State Pharmacopoeia of Ukraine using Microsoft Office Excel 2007 [35,50]. Differences between groups were statistically analysed using one-way analysis of variance (ANOVA). The results were expressed as mean ± standard deviation (SD). *p* values less than 0.05 were considered statistically significant.

## 4. Conclusions

Different ethanolic extracts from *G. aparine* herb were studied for its chemical composition and immunomodulatory activity. All ethanolic extracts from *G. aparine* herb significantly stimulated the transformational activity of immunocompetent blood cells, with 96% ethanolic extract being most active. The percentage of lymphocytes proliferating in RLBT under the influence of 96% ethanolic extract increased by 4.34–5.32 times compared with the spontaneous transformation. The results justify the traditional use of extracts from *G. aparine* as immunomodulatory agents.

The UHPLC-DAD-MS/MS analysis allowed comprehensive characterization of investigated extracts. Major phytochemicals from groups of polyphenols and iridoids were detected during the analysis. One flavonoid derivative namely quercetin 3-*O*-rhamnoglucoside-7-*O*-glucoside was isolated and identified for the first time in any *Galium* species investigated so far.

The data obtained give grounds for further research into the mechanisms of immunomodulatory activity of extracts from *G. aparine* herb.

## Figures and Tables

**Table 1:** The content of the main groups of phytochemicals in *Galium aparine* herb ethanolic extracts.

Extract	Extraction Yield (mg/mL)	Group of Phytochemicals (mg/g)			
		Polysaccharides	Hydroxycinnamic Derivates	Flavonoids	Polyphenols
Extract I (20% EtOH, *v*/*v*)	252.7 ± 12.6	129.4 ± 1.6	75.9 ± 0.5 *	10.7 ± 0.1 #	66.1 ± 0.5 *
Extract II (60% EtOH, *v*/*v*)	246.3 ± 12.3	n.d.	77.1 ± 0.6 *	10.2 ± 0.1 #	50.8 ± 0.6 #
Extract III (96% EtOH, *v*/*v*)	163.4 ± 8.1	n.d.	91.2 ± 0.5 #	15.3 ± 0.1 *	69.8 ± 0.5 *

*, # significant differences at *p* < 0.05, no significant differences observed for values with the same mark; n.d.—not detected.

**Table 2:** UHPLC-DAD-MS/MS data of compounds detected in analyzed extracts.

Peak No.	Compound Name	Retention Time (min)	UV (nm)	(M − H)^−^ *m*/*z*	MS^2^ ions	(M + H)^−+^ *m*/*z*	MS^2^ ions	Compound Content ug/mg		
								Extract I 20% EtOH	Extract II 60% EtOH	Extract III 96% EtOH
1	Monotropein^t^	2.1	237	389	369, 227, 209b, 183, 137	381	-	n.q.	n.q.	n.q.
2	10-Desacetylasperulosidic acid^t^	3.7	237	389	227b, 209, 183	381	-	n.q.	n.q.	n.q.
3	*p*-Hyroxybenzoic acid^s^	5.9	259, 294	153	-	155	-	n.q.	n.q.	n.q.
4	3-*O*-Caffeoylquinic acid^s^	9.7	217, 241, 300sh, 324	353	191b, 179, 161	355	163b	8.91 ± 0.09	3.72 ± 0.12	0.53 ± 0.03
5	Asperulosidic acid^t^	14.0	235	431	371, 269, 251b, 165	433	-	n.q.	n.q.	n.q.
6	5-*O*-Caffeoylquinic acid^s^ (Chlorogenic acid)	16.2	219, 241, 299sh, 325	353	191b, 179	355	163b	18.44 ± 0.21	31.51 ± 0.19	40.61 ± 0.12
7	4-*O*-Caffeoylquinic acid^s^	18.2	217, 241, 300sh, 325	353	191, 179, 173b	355	337, 307, 163b	8.12 ± 0.03	4.75 ± 0.08	1.16 ± 0.02
8	Quercetin 3-O-rhamnoglucoside-7-O-glucoside^s^	23.1	255, 263sh, 353	771	609b, 301	773	627, 611, 465b, 303	3.35 ± 0.10	2.95 ± 0.05	1.50 ± 0.02
9	Quercetin 3-O-rhamnoglucoside^s^ (Rutin)	36.9	255, 262sh, 354	609	591, 301b, 179	611	465, 303b	1.08 ± 0.05	2.49 ± 0.03	6.33 ± 0.07
10	Caffeic acid derivative	38.0	254, 299sh, 327	381	207, 191, 179b, 135	383	365, 163b	n.q.	n.q.	n.q.
11	Isorhamnetin 3-*O*-glucorhamnoside^s^	40.4	259, 260sh, 350	615	609b, 542, 461, 315	617	-	n.q.	n.q.	n.q.
12	3,4-*O*-Dicaffeoylquinic acid^s^	42.9	239, 300sh, 324	515	353b, 255, 173b	517	499b, 317, 163	n.q.	n.q.	n.q.
13	3,5-*O*-Dicaffeoylquinic acid^s^	43.8	240, 299sh, 324	515	353, 233, 191b, 179	517	499b, 147	0.23 +/− 0.02	0.42 +/− 0.08	2.43 +/− 0.11
14	4,5-*O*-Dicaffeoylquinic acid^s^	47.9	240, 300sh, 325	515	515, 353b, 299, 255, 203	517	499, 335b, 278	n.q.	n.q.	n.q.

s—comparisons with chemical standard have been made; b—base peak (the most abundant ion in recorded spectrum); sh-shoulder; t—tentative assignment; n.q.—not quantified.

**Table 3:** The effect of ethanolic extracts of *G. aparine* on the indices of lymphocyte blast transformation (X ± m), *n* = 5.

Extract	Extract Concentration (mg/mL)	RLBT, %
Extract ***I*** (20% EtOH, *v*/*v*)	0.10	32.4 ± 2.3 *
	0.74	35.2 ± 2.5 *
	1.47	34.7 ± 2.2 *
Extract ***II*** (60% EtOH, *v*/*v*)	0.10	34.6 ± 2.5 *
	0.74	38.5 ± 2.7 *
	1.47	36.8 ± 2.5 *
Extract ***III*** (96% EtOH, *v*/*v*)	0.10	36.9 ± 2.3 *
	0.74	45.2 ± 3,0 #
	1.47	45.1 ± 3.1 #
PHA	2.5	48.1 ± 2.1 #
Spontaneous RLBT	-	8.5 ± 0.7

PHA = phytohemagglutinin, RLBT = the reaction of lymphocyte blast transformation. *, #—*p* < 0.05 in comparison with PHA, different markers show statistically significant differences.
